# Advancing the monitoring of organelle contact sites *in vitro* and *in vivo*

**DOI:** 10.1042/BST20250371

**Published:** 2026-06-29

**Authors:** Lucia Barazzuol, Caterina Peggion, Marisa Brini, Yusuke Nasu, Tito Calì

**Affiliations:** 1 Department of Biomedical Sciences (DSB), University of Padova, Padova, Italy; 2 Department of Biology (DBio), University of Padova, Padova, Italy; 3 Department of Pharmaceutical and Pharmacological Sciences (DSF), University of Padova, Padova, Italy; 4 Study Center for Neurodegeneration (CESNE), University of Padova, Padova, Italy; 5 Institute of Biological Chemistry, Academia Sinica, Taipei, Taiwan; 6 Institute of Biochemical Sciences, National Taiwan University, Taipei, Taiwan; 7 Department of Chemistry, School of Science, The University of Tokyo, Tokyo, Japan; 8 Padova Neuroscience Center (PNC), University of Padova, Padova, Italy

**Keywords:** genetically encoded reporters, Organelle contact sites, SPLICS

## Abstract

Organelle contact sites are highly dynamic and specialized regions where distinct organelles come into proximity, enabling direct inter-organelle communication. These structures play fundamental roles in cellular homeostasis by coordinating the exchange of lipids, metabolites, and ions, as well as regulating key processes such as organelle dynamics, mitochondrial fission, autophagy, and metabolic integration. Alterations in contact site architecture and function have been increasingly associated with a wide range of human diseases, including neurodegeneration, metabolic disorders, and cancer. Despite their biological relevance, the nanoscale nature and dynamic behaviour of contact sites have historically posed significant challenges for their accurate detection and functional characterization.

Here, we provide a comprehensive overview of the methodologies currently available to study organelle contact sites, ranging from classical approaches such as electron microscopy and biochemical fractionation to advanced imaging techniques and genetically encoded reporters. We discuss recent developments in high-resolution and live-cell microscopy that have improved the spatial and temporal resolution of contact site analysis, as well as emerging tools designed to selectively label, quantify, and manipulate these interfaces. Attention is given to the next generation of engineered reporters capable of sensing molecular and ionic exchanges at contact sites, thereby moving beyond structural description toward functional interrogation. By critically evaluating the strengths and limitations of existing approaches, we aim to provide a framework for selecting appropriate tools and to highlight future directions in the field. Ultimately, advancing our ability to monitor and dissect organelle contact sites will be essential for understanding their contribution to cellular physiology and disease.

## Introduction

Organelle contact sites, also referred to as membrane contact sites (MCSs), are specialized regions where the membranes of two distinct organelles come into close proximity, typically within 10–30 nm, without undergoing membrane fusion. These structures are stabilized by specific tethering proteins or protein complexes that bridge the opposing membranes, allowing the formation of dynamic and regulated interfaces between organelles. Organelle contact sites have been identified between virtually every intracellular compartment, including endoplasmic reticulum (ER), mitochondria, lysosomes, endosomes, peroxisomes, lipid droplets (LDs), and Golgi apparatus and function as hubs for inter-organelle communication. They facilitate the direct exchange of lipids, metabolites, and ions, for example enabling cells to coordinate metabolic pathways that span multiple organelles [1]. These interfaces also participate in the regulation of organelle dynamics, including mitochondrial fission, autophagosome formation, and organelle biogenesis [2,3]. As such, organelle contact sites provide an efficient means of integrating many different processes, becoming central in maintaining cellular homeostasis [4]. Rather than serving as static structural connections, organelle contact sites have a dynamic nature that allows cells to rapidly remodel inter-organelle communication in response to physiological demands.

Given their central role in cellular homeostasis, it is not surprising that dysregulation of organelle contact sites has been increasingly linked to human diseases. Alterations in ER–mitochondria contacts have been associated with neurodegenerative disorders, including Alzheimer’s disease, Parkinson's disease, and amyotrophic lateral sclerosis, where disrupted calcium signaling and mitochondrial dysfunction contribute to neuronal damage [1,5–9]. Similarly, defects in contact sites involving lysosomes, peroxisomes, and LDs have been implicated in metabolic diseases, lipid storage disorders, and cancer [10–13]. Pathogens have also evolved mechanisms to exploit or remodel organelle contact sites to facilitate their replication within host cells [14].

Understanding the molecular mechanisms that govern the formation, regulation, and function of these contacts is therefore crucial not only for elucidating basic cell biology but also for uncovering new therapeutic strategies for diseases linked to organelle dysfunction. In this context, the availability of appropriate methods to accurately monitor organelle contact sites is of paramount importance. Here, we will specifically focus on these aspects, discussing the tools currently available and commonly used to study contact sites, as well as their strengths and limitations. We will also highlight recent advances in engineering these approaches to increase their complexity and enable the sensing of molecules and ions at contact sites.

## Classical investigation methods

Organelle contact sites were first discovered using classical cell biology techniques. Electron microscopy (EM) provided the earliest direct images of these close membrane interactions [15] and enabled measurement of their distance. EM remains the ‘gold standard’ for confirming contacts between organelles, even though it is limited to fixed cells. In parallel with ultrastructural studies, biochemical approaches, such as subcellular fractionation, helped identify the molecular composition of these sites, revealing enrichment in specific proteins and lipids involved in processes like lipid synthesis [16,17]. Conventional fluorescence microscopy also contributed to the study of organelle interactions, even though its spatial resolution is limited by the diffraction limit of light (approximately 200–250 nm), which is much larger than the actual distance between contacting membranes. By labeling different organelles with fluorescent markers, researchers could observe regions where the signals from two organelles appeared closely apposed or partially overlapping, suggesting the presence of contact sites. Importantly, fluorescence microscopy allowed live-cell imaging, providing insights into the dynamic nature of organelle interactions and showing that many contacts form and dissolve over time. Finally, immuno-EM combined structural detail with protein localization, helping identify the molecules that tether organelles together. Together, these classical techniques laid the foundation for our understanding of how organelles communicate within the cell. However, because organelle contact sites are highly dynamic and often transient, classical approaches are limited in their ability to fully capture their formation, remodelling, and dissolution over time. This highlighted the need for the development of more advanced techniques capable of resolving these interactions with greater spatial and temporal precision, in order to better understand their regulation and functional significance within living cells.

## Advanced investigation methods

**Figure 1:**
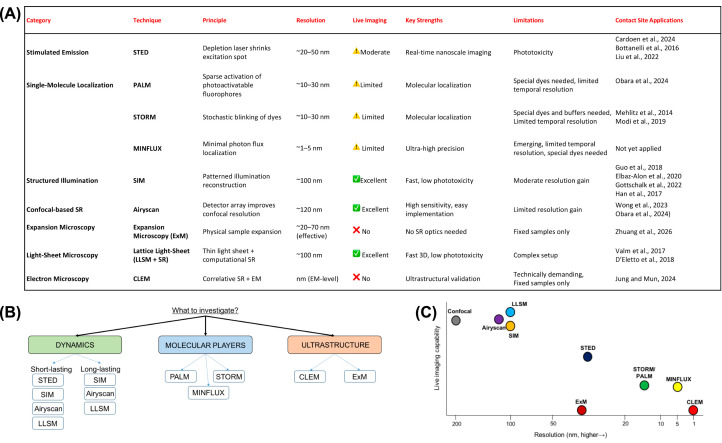
Imaging techniques to investigate organelle contact sites (**A**) Schematic overview of newly developed imaging techniques. (**B**) Strategy for selecting high/super-resolution microscopy techniques to investigate organelle contact sites. Techniques are grouped based on their primary application: live-cell dynamics, molecular composition, and ultrastructural validation. No single method captures all dimensions; therefore, complementary approaches are typically required. (**C**) Schematic representation of resolution versus live imaging capability of the different imaging techniques.

One of the drawbacks of fluorescence microscopy, as previously stated, is its diffraction limit, preventing the visualization of the contacts at the right nanometer scale. Because MCS typically involve intermembrane distances of only ∼10–30 nm, in fact, they are difficult to resolve using traditional fluorescence microscopy. To address this problem, technological advances have followed two main directions: overcoming the optical limitations of conventional microscopy techniques, and the creation of specialized reporters designed to specifically label and detect contact sites (Figure 1A).

### New microscopy techniques

The investigation of MCSs has greatly benefited from the development of super-resolution microscopy techniques, which overcome the diffraction limit of conventional fluorescence microscopy. Advanced imaging approaches such as stimulated emission depletion microscopy, structured illumination microscopy, and single-molecule localization techniques, including photoactivated localization microscopy and stochastic optical reconstruction microscopy, have therefore become essential tools for studying these structures [18–24]. Their spatial, ∼20–50 nm (x–y), ∼50–150 nm (z); ∼100 nm (x–y), ∼250–300 nm (z); ∼10–30 nm (x-y), ∼20–60 nm (z, 3D implementations), and temporal resolution, milliseconds to seconds (live-cell compatible); up to ∼1–10 fps in live imaging and seconds to minutes, respectively, allow researchers to visualize the organization of contact site components, determine the nanoscale distribution of tethering proteins, and examine how contact sites form, remodel, and dissolve in living or fixed cells with unprecedented spatial and temporal resolution. Instead of observing broad overlapping fluorescence signals, researchers can now distinguish closely apposed membranes and measure the relative positioning of proteins located at contact interfaces. This improvement, for instance, has enabled the identification of nanoscale domains within contact sites and has revealed that these structures are often highly heterogeneous rather than uniform along organelle surfaces [24]. Consequently, super-resolution microscopy is playing an increasingly important role in elucidating the molecular architecture and functional dynamics of MCS in both physiological and pathological contexts. See (Figure 1) for a specific description of the different super-resolution techniques.

Airyscan microscopy is an advanced imaging technique well suited for live-cell imaging with a spatial resolution of ∼120–140 nm (x–y), ∼300–400 nm (z) and temporal resolution of milliseconds to seconds. Although it does not reach the resolution of EM or some super-resolution optical methods, it significantly improves the ability to detect and quantify contact regions using standard fluorescent labeling [25]. This makes it especially valuable for studying the distribution and organization of contact sites across whole cells. A key advantage of Airyscan microscopy is its compatibility with live-cell imaging. Its increased sensitivity reduces photobleaching and phototoxicity, enabling the observation of dynamic processes such as the formation, remodelling, and dissolution of organelle contacts in real time [21,24,26].

In the study of organelle contact sites, light sheet fluorescence microscopy (LSFM) is especially valuable for capturing their dynamic behaviour over time in intact cells or even whole organisms (spatial resolution of ∼200–400 nm (x–y), ∼500–800 nm (z) and temporal resolution of milliseconds to seconds (high-speed volumetric imaging)). It is a powerful imaging technique that enables fast, high-contrast visualization of biological samples by illuminating them with a thin sheet of light. Although its spatial resolution is generally lower than that of super-resolution techniques, LSFM excels at imaging large volumes with high temporal resolution, enabling researchers to follow the formation, movement, and remodelling of organelle interactions in a physiological context [21,27].

Expansion microscopy (ExM) has emerged as a powerful parallel approach that physically enlarges biological samples, rather than relying solely on optical improvements [28], it has a spatial resolution of ∼60–70 nm (x–y) with ∼4× expansion (down to ∼20–30 nm with higher expansion), ∼150–200 nm (z, improved with expansion) and no temporal resolution since it is for fixed samples only. In this method, cells or tissues are embedded in a swellable polymer that isotopically expands after processing, effectively increasing the distance between molecules while preserving their relative spatial organization. This allows conventional microscopes to achieve nanoscale resolution, making it possible to overcome their diffraction limit. In the context of investigation for organelle contact sites, ExM was recently refined into a novel labeling technique termed landscape ExM (land-ExM), which achieves high signal-to-noise ratios for both protein and lipid imaging. Its application was recently applied to visualize 3D interactions between membrane-bound organelles and phase-separated condensates, uncovering triple-organelle contact sites among stress granules, nuclear tunnels and adjacent nucleoli [29].

The multispectral imaging approach allows the simultaneous visualization of multiple organelles and their interactions within the same cell the spatial resolution of ∼200–300 nm (x–y), ∼500–700 nm (z) and temporal resolution of seconds (it enables multiplexed organelle imaging). This strategy enables a more global view of how different compartments are spatially organized and reveals how multiple contact sites coexist, potentially coordinate with each other and how relationship between organelles evolve over time (Figure 1B,C). The method is based on the use of a palette of spectrally distinct fluorescent proteins, each targeted to a different organelle. By carefully selecting fluorophores with minimal spectral overlap and combining them with advanced imaging systems capable of spectral unmixing, it becomes possible to image 6 organelles within the same cell. This overcomes a major limitation of traditional fluorescence microscopy, where only two or three organelles could typically be imaged at once [27,30,31].

### New specialized reporters

**Figure 2:**
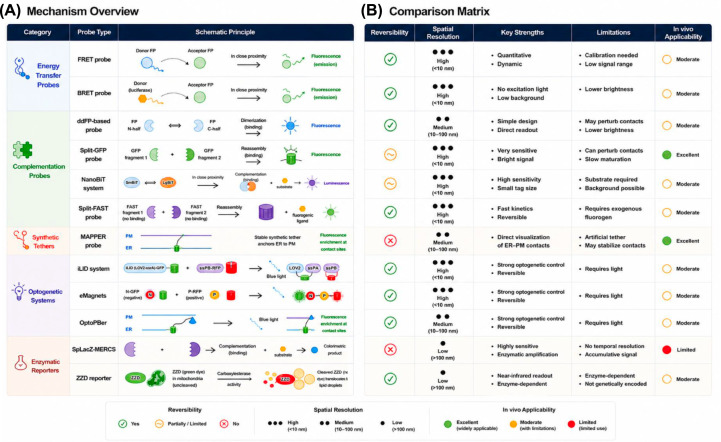
Genetically encoded reporters to detect and manipulate organelle contact sites Comparative overview of the main classes of probes used to detect and manipulate organelle contact sites, categorized according to their underlying detection mechanism and features. (**A**) Mechanism overview. Schematic representations of the working principles of major probe classes. (**B**) Comparison matrix. Summary of probe properties including reversibility, spatial resolution, signal type, advantages, limitations, and *in vivo* applicability.

As for the development of specialized reporters for contact sites, various strategies have been employed to achieve the goal. The following paragraphs aim to provide the comprehensive summary of the available tools (Figure 2).

The principle of Förster resonance energy transfer (FRET) has been used to detect extremely close proximity between two organelles [32–36]. In these systems, two different fluorophores are targeted to separate membranes, typically a donor such as blue fluorescence protein (FP) and cyan FP, and an acceptor such as green FP (GFP) and yellow FP (YFP). When the organelles approach at nanoscale proximity, FRET occurs from the donor to the acceptor upon excitation, producing a measurable shift in fluorescence. Because this process is highly distance- and orientation-dependent and reversible, FRET probes are particularly well suited for monitoring rapid and transient contact events in real time, although their signals can be relatively weak and require careful calibration. The system has been further improved, first by adding a chemically inducible dimerization portion responsive to rapamycin or its analogues so called rapalogs. Administration of these chemicals can induce contact sites at organelles of interest and their effect on downstream cellular responses can be monitored. Another modified FRET probe incorporates a Pericam Ca^2+^-sensing domain, consisting of calmodulin fused to its target peptide M13, which is derived from the CaM-binding region of the skeletal muscle myosin light-chain kinase. Its fluorescence changes in response to the Ca^2+^ dependent interaction between CaM and M13, allowing the monitoring of local Ca^2+^ fluxes that occur at organelle interfaces [32,37]. Because Ca^2+^ transfer is a key functional hallmark of many contact sites, these probes provide an indirect but relevant readout of contact activity rather than just proximity.

Bioluminescence resonance energy transfer (BRET) probes represent an alternative to FRET-based approaches. Like FRET, BRET relies on energy transfer between a donor and an acceptor when they are in very close proximity: however, instead of using external light excitation for an FP, BRET uses a bioluminescent enzyme (such as luciferase) as the donor, which emits light upon substrate addition, exciting the acceptor FP. The MERLIN (mitochondria–ER length indicator) is a BRET-based probe specifically designed to monitor the distance between ER and mitochondria: to generate this probe, a luciferase with larger brightness and stability was used to enhance signal intensity [38]. In fact, the bioluminescence signals from BRET probe is generally weaker than the fluorescence signal from FRET probes, and depend on substrate availability and luciferase activity, which can complicate quantitative analysis. A BRET-based Ca^2+^ probe has been engineered by incorporating a Ca^2+^-sensing domain into the interface between luciferase and FP. For example, the mitochondria-associated ER membrane (MAM)-Calflux tool has been applied to investigate ER–mitochondria contact sites, enabling parallel structural and functional characterization of this interorganelle interface and representing a versatile tool to detect Ca^2+^ fluxes between the two organelles [39].

Another luciferase-based system applied for investigating organelle contact sites is the NanoBiT, a split-luciferase complementation system derived from NanoLuc luciferase (Promega). The NanoBiT system consists of two fragments: a large fragment (LgBiT, ∼18 kDa) and a small fragment (SmBiT, 11 amino acids) [40]. They come together to reconstitute a functional enzyme, producing a luminescent signal in the presence of luciferase substrate. The interaction between LgBiT and SmBiT is reversible, and the system can be used to detect rapidly dissociating interactions. Recently, MiMSBiT (mitochondria–melanosome contact reporter using NanoBiT) exploited the system to investigate mitochondria–melanosomes contact sites, allowing the characterization of some of the players involved in this communication [41]. The system has also been applied to measure the reversibility of ER–mitochondria contact sites formation in living cells, thereby revealing the key role of these contacts in cellular survival strategies [42].

Split-FP-based probes are a widely used class of genetically encoded tools that rely on the reconstitution of the FP from two non-fluorescent fragments. The 11-stranded β-barrel GFP is split into two parts: 1–10 β strands (GFP1-10) and 11th β strand (GFP11) or 1–8 and 9–11 β strands, one of the earliest split-FP based bimolecular fluorescence complementation (BiFC) strategies and has been applied to study protein–protein interactions in cells [43,44]. Individually, these fragments are not fluorescent, but they reassemble into a fully folded fluorescent FP molecule upon close proximity. For contact site studies, the two FP fragments are targeted to different organelles: when these approach, the FP fragments complement and produce fluorescence specifically at their contacts. Variants of these reporters have been engineered with different linker lengths, allowing detection of either very tight or more extended contacts, and targeting different organelle pairs [45–48]. Recently, a split version of the mNeonGreen FP was engineered to simultaneously monitor ER–mitochondria contact sites and their mediated Ca^2+^ fluxes, again allowing parallel structural and functional investigations of the organelle interface [49]. Split FPs have been applied to the investigation of various organelle contact sites [50–52] and have also been tested *in vivo* in whole organisms such as *Danio rerio* and *Drosophila melanogaster*. These probes are highly effective for visualizing the spatial distribution of contact sites in living cells, especially for their bright fluorescence; although the complementation of the system has been thought to be poorly reversible, consequently stabilizing contact sites *per se*, recent evidence showed reversibility in cell lines and *in vivo* [48,53]. A recent paper, for example, applied the system to study ER–Golgi contact sites and reported individual puncta undergoing rapid expansion, shrinkage, coalescence, and splitting within seconds, further confirming the *in vivo* reversibility of the split GFP system [54,55].

Dimerization-dependent FP (ddFP)-based reporters are a class of genetically encoded tools, consisting of two non-fluorescent FP variants expressed separately and targeted to different organelles [26,56–60]. The two FPs dimerize to fluoresce upon the distance between the membranes in a reversible manner. A range of color variants have been engineered for the generation of ddFP-based probes: ddGFP and ddYFP [60], ddRFP [26,59], allowing the simultaneous labeling of two distinct contact sites. Unlike canonical split FPs discussed above, ddFP system enables to detect both the formation and dissociation of contact sites with higher temporal resolution. Therefore, ddFP probes provide a useful compromise between spatial and temporal resolution, although their fluorescence intensity is generally lower than that of the original parental FPs.

FAST (Fluorescence-activating and absorption-shifting tag) is a small, engineered protein tag (∼14 kDa) that specifically binds to a cell-permeable ligand [61]. The ligand is fluorescent only when it is bound to FAST protein, allowing low background fluorescence signal. In the split-FAST system [62], the FAST protein is divided into two fragments. These fragments are genetically fused to different target proteins. When the two fragments come into close proximity, they reassemble into a functional FAST domain capable of binding the fluorogenic ligand and generating a fluorescence signal. This system has been applied to investigate ER-mitochondria and ER–plasma membrane (PM) contacts [21], and recently to the creation of a tool kit (FABCON) that can be used to uncover the dynamic regulation of the interaction of LD with any other organelle [63].

The genetically encoded reporter MAPPER (membrane attached peripheral ER) selectively labels ER–PM junctions based on the subcellular targeting mechanisms of STIM1 [64]. STIM1 is a single transmembrane ER protein that localizes with its N terminus in the ER lumen and C terminus in the cytosol. Upon ER Ca^2+^ depletion, STIM1 oligomerizes and translocate to ER–PM junctions, where it binds to PM phosphoinositides thanks to it C-terminal polybasic motif. The MAPPER probe consists of GFP targeted to the luminal face of the ER through the STIM1 signal peptide and transmembrane domain at the N and C termini, respectively. To enable constitutive localization at ER–PM junctions, the probe was further engineered to protrude from the ER surface by fusion with the polybasic motif of the small G protein Rit, which constitutively binds to phosphoinositides in the PM. Consequently, this probe interacts electrostatically with phosphoinositides in the inner leaflet of the PM, allowing the visualization of ER–PM contact sites [65,66].

The SpLacZ-MERCs probe is a genetically encoded reporter designed to detect and study mitochondria–ER contact sites (MERCs) by exploiting a split β-galactosidase enzyme (LacZ) complementation system. Simply, the LacZ is divided into two inactive fragments which are targeted to the surfaces of the two organelles respectively. These fragments reassemble upon close proximity to reconstitute β-galactosidase activity at organelle contact sites after the administration of the enzyme substrate [67]. Although this system does not permit visualization of exact contact sites, it enables a novel high-throughput screening strategy for the identification of molecules/genes that regulates MERCS levels.

The ZZD reporter is a recently published dual-organelle-targeted near-infrared multifunctional fluorescent dye [68]. This near-infrared fluorescent molecule targets mitochondria thanks to its positive charge. However, upon hydrolysis by carboxylesterases (CEs), key enzymes that hydrolyse triglycerides in LDs, such probe generates a hydrophobic, uncharged fluorophore that locates into LDs, showing a high selectivity and a rapid response (within 20 s), accompanied by the emission of a green fluorescence signal. This dye enables simultaneous dual-organelle imaging of mitochondria and LDs to track their dynamic interactions in real time. It has been developed for the investigation of acute alcoholic liver injury, a critical early stage in the progression of alcoholic liver disease characterized by decreased CE activity, abnormal lipid accumulation, and mitochondrial dysfunction. Functioning as a near-infrared probe, it offers advantages such as low background noise, high photostability, and reduced photodamage for both live-cell and in mouse model studies.

Different optogenetic dimerization systems were exploited for the study of contact sites. iLID (improved light-inducer dimer) consists of two main parts: the iLID domain, a modified version of the LOV2 (light-oxygen-voltage) domain from Avena sativa phototropin 1, fused to a small peptide called SsrA; the binding partner (SspB), a protein domain that binds specifically to the SsrA peptide when the LOV2 domain is activated by light. In the dark, the SsrA peptide is caged within the LOV2 domain, preventing SspB binding. Upon exposure to blue light (∼450–488 nm), LOV2 undergoes a conformational change that exposes SsrA, allowing SspB to bind. This interaction is reversible: when the light is turned off, the LOV2 domain relaxes and SspB dissociates. By attaching the two parts of iLID to the surfaces of two organelles, the system can be used to induce or stabilize organelle interactions in cells [69].

Enhanced Magnets (eMagnets) are another optogenetic dimerization system, similar in concept to iLID. eMagnets are based on engineered versions of the LOV domain. Two engineered LOV-derived domains are designed as complementary pairs: one domain is called ‘magnets-positive’ (+) and the other is ‘magnets-negative’ (−). In the dark, they have minimal affinity for each other. Upon blue-light exposure, conformational changes expose the dimerization interface, driving heterodimer formation. The effectiveness of this system was confirmed in a variety of application, including the investigation of mitochondria–lysosomes, ER–mitochondria, and ER–lysosomes contact sites generation/expansion [70].

OptoPBer is a genetically encoded, optogenetic reporter and manipulator designed to study ER–PM contact sites. It is an optogenetic-version of the MAPPER reporter: it incorporates a LOV2 domain, that only upon blue light stimulation allows the exposure of the polybasic region of the probe, mediating the interaction with the PM [71].

When using optogenetic reporters, it is important to consider that contacts are induced upon light illumination, so that could be a disadvantage when investigating MCS dynamic in physiological conditions.

The *in situ* proximity ligation assay (PLA) is used to convert the physical closeness of two proteins into a detectable molecular signal inside fixed cells [72]. The principle relies on selecting one protein resident on each organelle—for example, a mitochondrial outer membrane protein and an LD-associated protein—and probing them with two different antibodies. When these proteins come within nanometer-scale distance, as occurs at contact sites, the DNA-tagged secondary antibodies used in PLA are brought close enough to allow ligation of their attached oligonucleotides into a circular DNA molecule. This circular DNA is then locally amplified by hybridization with fluorophore-labeled oligonucleotides and visualized as a bright fluorescent spot, effectively marking the position of a contact event. In practice, PLA provides a spatial map of contact frequency and distribution across the cell and has been optimized to study different contacts and in different experimental set up [73–75]. However, the assay captures a fixed snapshot, so it cannot resolve the dynamics or duration of individual contacts. It also depends on the choice of marker proteins, meaning that what is detected reflects proximity between those specific molecular components rather than the entirety of the contact interface.

See (Figure 2) for a comprehensive recapitulation of the available specialized reporters and their features. Despite significant effort and the wide, up-to-date range of available probes, the field still lacks a universally applicable tool that combines all the capabilities of currently known reporters. As a result, researchers must carefully select the probe that best addresses their specific scientific question. Furthermore, the functional specificity of contact sites remains poorly understood, highlighting the need for further engineering approaches that can integrate proximity detection with the analysis of activity at membrane interfaces.

## EM development

It is also worth mentioning the development of EM, which is still considered a gold standard when working with contact sites. However, standard EM techniques, on top of requiring a fixed sample, rely on the chemical fixation and dehydration of samples, thus potentially altering their ultrastructure. Advances in EM based on cryofixation have overcame this problem by allowing biological samples to be preserved in a near-native state without the distortions introduced by chemical fixation, dehydration, or staining. Rapid freezing immobilizes molecules within milliseconds, trapping membranes, protein complexes, and organelle interfaces exactly as they exist in living cells. This has been particularly important for studying delicate and dynamic structures such as MCSs, whose nanoscale organization is highly sensitive to preparation artefacts. Techniques such as cryo-electron tomography enable three-dimensional reconstruction of cellular regions at nanometer resolution directly inside intact cells [76]. The introduction of cryo-focused ion beam milling (cryo-focused ion beam scanning electron microscopy—cryo-FIB-SEM) has further expanded this capability by allowing thick cellular specimens to be thinned into lamellae suitable for tomography, making it possible to access interior regions of cells that were previously inaccessible. Cryofixation has also enabled the direct visualization of macromolecular assemblies at contact sites, bridging the gap between structural biology and cell biology [77,78]. Another major advance is the integration of cryo-electron microscopy with correlative light microscopy (cryo-CLEM), which allows fluorescently labeled structures to be located first and then imaged at high resolution by EM. This combination enables researchers to connect dynamic or functional information with ultrastructural detail in the same sample [79].

## Biochemical methods

In addition to imaging-based methods, biochemical strategies have evolved to dissect contact site compositions. In particular, proximity labeling (PL) approaches: they are used to study organelle contact sites and they all rely on the same core idea, an engineered enzyme generates a short-lived reactive molecule that tags nearby proteins, even though they differ substantially in kinetics, spatial precision, and how specifically they restrict labeling to the actual interface between two organelles.

The original BioID method is based on a mutated biotin ligase (BirA*) that slowly produces a reactive biotin intermediate capable of covalently modifying nearby lysine residues. Because labeling requires many hours, it effectively integrates interactions over long periods, leading to significant background labeling and reducing spatial and temporal resolution. TurboID and its smaller variant miniTurbo were developed to overcome this limitation by dramatically increasing catalytic efficiency [80], completing labeling within minutes. Although they still label within a similar spatial radius, the shorter labeling window reduces nonspecific accumulation. APEX and its improved version APEX2 take a different enzymatic approach, relying on an engineered peroxidase that generates highly reactive radicals in the presence of hydrogen peroxide. These radicals react almost instantly with nearby proteins, giving the method an exceptional temporal resolution on the order of seconds. At the same time, the requirement for hydrogen peroxide introduces potential cellular stress, and the reactive species can diffuse slightly farther than the BioID-derived intermediates, which may modestly reduce spatial precision [81].

A major conceptual advance came with the development of split-enzyme systems such as split-BioID [82], split-TurboID [83,84], and split-APEX, splitted versions of the enzymes [81]. This design greatly enhances specificity and minimizes background from proteins residing on individual organelles, making these methods particularly valuable for defining the proteome of bona fide contact sites rather than general organelle neighbourhoods.

More recently, hybrid systems such as BiFC–PL have been introduced to integrate PL with imaging. In these methods, BiFC is used to bring together two fragments targeted to different organelles. When contact occurs, fluorescence is restored and PL is activated simultaneously. This dual functionality allows researchers to both visualize contact sites in living cells and identify their molecular components, providing a powerful link between spatial observation and proteomic analysis [85]. Such approach was used to study either MERCs or mitochondria–LD contacts and allowed the identification of 403 highly confident MERC proteins. A related approach worth mentioning is the CsFiND system (complementation assay using fusion of split-GFP and TurboID), which enables simultaneous visualization of MCSs and PL of MCS-associated proteins in yeast [86]. The practical utility of this approach has recently been demonstrated through its application to the identification of novel MCS-associated proteins involved in the yeast nucleus–vacuole junction [87]. Another method called ABOLISH (Auxin-induced BiOtin LIgase diminiSHing) was developed to study stable and transient interactions in yeast. This approach was designed to reduce the high endogenous background biotinylation of proteins in yeast, thereby enhancing signal detection from exogenous biotin ligases [88].

A particularly advanced development is OrthoID, which introduces the concept of orthogonal PL. Instead of relying on a single enzyme, this method uses two distinct labeling systems operating in parallel, each targeted to a different organelle (es: turbo-ID and APEX). Because the two labeling chemistries are independent, proteins can be analyzed based on whether they carry one tag or both. Proteins labeled by only one system are likely associated with a single organelle, whereas proteins that are labeled by both are strong candidates for being located at the contact interface. This ‘intersection’ strategy greatly improves specificity and allows a more refined definition of contact site proteomes. However, the method is experimentally more complex, as it requires the coordination of two labeling systems and more sophisticated data analysis [89].

Overall, the field is shifting toward approaches that combine multiple layers of control, including enzyme splitting, orthogonal chemistries, and inducible activation.

## Conclusions

In recent years, methodological advances have profoundly expanded our ability to study organelle contact sites, moving the field from static observations to increasingly dynamic and mechanistic understanding. Despite these advances, many aspects of organelle communication remain difficult to capture, particularly their transient nature, molecular heterogeneity, and functional diversity across cellular contexts. Continued development of more sensitive, specific, and integrative tools will therefore be essential to fully resolve the complexity of these structures. Such progress will be critical not only for understanding fundamental cell biology but also for elucidating the role of organelle contact sites in physiology and disease.

## Perspectives

- **Importance of the field:** Organelle contact sites (MCSs) have emerged as fundamental regulators of cellular organization and homeostasis, acting as hubs for inter-organelle communication. By coordinating the exchange of lipids, metabolites, and ions, and by controlling processes such as mitochondrial dynamics, autophagy, and metabolic integration, these structures are central to cell physiology. Importantly, their dysregulation has been increasingly linked to a wide spectrum of human diseases, including neurodegeneration, metabolic disorders, and cancer, highlighting their relevance as potential therapeutic targets.
- **Current thinking:** The field has evolved from viewing contact sites as static structural connections to recognizing them as highly dynamic and heterogeneous platforms with specialized functions. Significant advances in imaging technologies and biochemical approaches have enabled the identification of molecular components and the visualization of contact site architecture at increasing resolution. More recently, the development of genetically encoded reporters and super-resolution microscopy has begun to reveal the spatial organization and temporal dynamics of these interfaces. However, current methodologies still present limitations, particularly in linking structural observations to functional outputs such as molecular and ionic exchange.
- **Future directions:** Future progress in the field will depend on the development of next-generation tools capable of integrating structural and functional information. In particular, engineered reporters that can directly sense molecular and ionic fluxes at contact sites will be critical to move beyond descriptive analyses toward mechanistic understanding. Advances in live-cell and volumetric imaging, multiplexed approaches, and quantitative methods will further enable the study of contact sites in complex and physiological contexts. Ultimately, combining these technologies with genetic and biochemical perturbations will provide new insights into how contact site dysfunction contributes to disease and may open new avenues for therapeutic intervention.

## Open Access

Open access for this article is provided via our Subscribe to Open programme, made possible through the support of our subscribing institutions. Learn more here: https://portlandpress.com/pages/open_access_options_and_prices#conditional.

## Abbreviations

BiFC: bimolecular fluorescence complementation
BRET: bioluminescence resonance energy transfer
CE: carboxylesterase
ddFC: dimerization-dependent FP
EM: electron microscopy
ExM: expansion microscopy
FAST: fluorescence-activating and absorption-shifting tag
FP: fluorescence protein
FRET: Förster resonance energy transfer
GFP: green FP
iLID: improved light-inducer dimer
LSFM: light sheet fluorescence microscopy
MCS: membrane contact site
MERC: mitochondria-ER contact site
PL: proximity labelling
PLA: proximity ligation assay
PM: plasma membrane
YFP: yellow FP
SPLICS: split-GFP-based contact site sensor
